# Linkage Mapping and Genome-Wide Association Studies of the *Rf* Gene Cluster in Sunflower (*Helianthus annuus* L.) and Their Distribution in World Sunflower Collections

**DOI:** 10.3389/fgene.2019.00216

**Published:** 2019-03-14

**Authors:** Zahirul I. Talukder, Guojia Ma, Brent S. Hulke, Chao-Chien Jan, Lili Qi

**Affiliations:** ^1^Department of Plant Sciences, North Dakota State University, Fargo, ND, United States; ^2^Edward T. Schafer Agricultural Research Center, Agricultural Research Service, United States Department of Agriculture, Fargo, ND, United States

**Keywords:** sunflower, restorer-of-fertility, downy mildew, linkage mapping, genome-wide association study, gene cluster

## Abstract

Commercial hybrid seed production in sunflower currently relies on a single cytoplasmic male sterility (CMS) source, PET1 and the major fertility restoration gene, *Rf1*, leaving the crop highly vulnerable to issues with genetic bottlenecks. Therefore, having multiple CMS/*Rf* systems is important for sustainable sunflower production. Here, we report the identification of a new fertility restoration gene, *Rf7*, which is tightly linked to a new downy mildew (DM) resistance gene, *Pl_34_*, in the USDA sunflower inbred line, RHA 428. The *Rf7* gene was genetically mapped to an interval of 0.6 cM on the lower end of linkage group (LG) 13, while *Pl_34_* was mapped 2.1 cM proximal to the *Rf7*. Both the genes are located in a cluster of *Rf* and *Pl* genes. To gain further insights into the distribution of *Rf* genes in the sunflower breeding lines, we used a genome-wide association study (GWAS) approach to identify markers associated with the fertility restoration trait in a panel of 333 sunflower lines genotyped with 8,723 single nucleotide polymorphism (SNP) markers. Twenty-four SNP markers on the lower end of LG13 spanning a genomic region of 2.47 cM were significantly associated with the trait. The significant markers were surveyed in a world collection panel of 548 sunflower lines and validated to be associated with the *Rf1* gene. The SNP haplotypes for the *Rf1* gene are different from *Rf5* and the *Rf7*gene located in the *Rf* gene cluster on LG13. The SNP and SSR markers tightly flanking the *Rf7* gene and the *Pl_34_* gene would benefit the sunflower breeders in facilitating marker assisted selection (MAS) of *Rf* and *Pl* genes.

## Introduction

Cytoplasmic male sterility (CMS) is a maternally transmitted trait carried by the mitochondrial genome that prevents hermaphrodite plants from developing viable pollen, resulting in male-sterile plants (Chase, 2007). CMS provides a very useful mechanism to produce large numbers of female plants for commercial hybrid seed production (Harvey, 2004). CMS is a common phenomenon observed in over 150 diverse plant species (Laser and Lersten, 1972; Schnable and Wise, 1998; Fujii et al., 2011). In populations with CMS, one or more nuclear genes known as restorer-of-fertility (*Rf*) genes can suppress the expression of the aberrant mitochondrial CMS genes and restore viable pollen production (Chase, 2007). The CMS/*Rf* system has been an indispensable resource for commercial hybrid seed production in many crops including sunflower (Bohra et al., 2016).

The first CMS source of sunflower, PET1, was derived from an interspecific cross between *Helianthus petiolaris* subsp. *petiolaris* Nutt. and cultivated sunflower (Leclercq, 1969). Soon after, Kinman (1970) discovered a single dominant gene, *Rf1*, in the sunflower line T660006-2-1 that restores the fertility of CMS PET1 (Supplementary Figure S1). Since then, the *Rf1* gene has been introduced from T660006-2-1 to many public and private sunflower inbred lines (Korell et al., 1992). A second dominant gene, *Rf2*, complementary to *Rf1* was discovered from a restorer line MZ-1398 (Vranceanu and Stoenescu, 1971, 1978). However, *Rf2* was described to be ubiquitous in nearly all sunflower inbred lines, including maintainer lines of PET1 (Horn et al., 2003; Serieys, 2005); therefore, *Rf1* is the most important gene to track for fertility restoration of CMS PET1. Since the first discovery of CMS PET1, there have been over 70 CMS sources reported in sunflower (Serieys, 2005). Many of these CMS sources do not have a known complementary *Rf* gene or have an unstable cytoplasm (Seiler and Rieseberg, 1997).

To date, only seven *Rf* genes have been characterized and mapped in sunflower genome. The *Rf1* gene from the sunflower lines RHA 266, RHA 271, RHA325, and RHA 439 was mapped to LG13 by different researchers (Gentzbittel et al., 1995; Berry et al., 1997; Horn et al., 2003; Kusterer et al., 2005; Yue et al., 2010). Gentzbittel et al. (1995, 1999) reported the presence of a distinct fertility restoration locus, *Msc1*, for the CMS PET1 cytoplasm and mapped it to LG12 on the restriction fragment length polymorphism (RFLP) map, which corresponds to LG7 of the public map. The *Rf3* gene mapped on LG7 in the inbred lines RHA 280 and RHA 340 is equally capable of restoring fertility of PET1 CMS lines (Jan and Vick, 2007; Abratti et al., 2008; Liu et al., 2012). Feng and Jan (2008) identified a new dominant restorer gene, *Rf4* originally from the diploid perennial wild species *H. maximiliani*, and mapped it to LG3 of the sunflower genome. The *Rf4* gene restored the pollen fertility of the CMS GIG2 cytoplasm derived from wild *H. giganteus*. Schnabel et al. (2008) mapped the *Rf-PEF1* gene for CMS PEF1 cytoplasm and demonstrated that *Rf-PEF1* is not located on LG13 where the *Rf1* gene resides. A restorer gene designated as *Rf5* derived from wild *H. annuus* was mapped to the lower end of LG13 close to the *Rf1*gene (Qi et al., 2012). The most recent *Rf* gene described in sunflower, *Rf6*, was derived from *H. angustifolius* and is required to restore the male fertility of CMS 514A developed recently with *H. tuberosus* cytoplasm (Liu et al., 2013). *Rf6* was mapped to LG3 of the public sunflower map.

While *Rf* genes have been mapped throughout the genome in different crop species, clustering of multiple *Rf* genes in the genome is also common across species (Melonek et al., 2016). For example, *Rf1* and *Rf5* genes were mapped in similar position on LG13 in sunflower (Yue et al., 2010; Qi et al., 2012), and in rice *Rf1a* (*Rf5*), *Rf1b*, *Rf4*, and *Rf6(t)* were mapped in proximity on chromosome 10 (Huang et al., 2000; Liu et al., 2004; Ahmadikhah and Karlov, 2006; Wang et al., 2006; Hu et al., 2012). In sunflower, the PET1 cytoplasm and its corresponding *Rf1* gene has been solely used by the commercial seed industries around the globe for large-scale hybrid seed production (Dimitrijevic and Horn, 2018). Use of a single CMS source in sunflower hybrid production carries the risks associated with genetic bottlenecks in crops. The southern corn leaf blight epidemic of T-cytoplasmic maize revealed the dangers of hybrid-seed production using a single source of CMS (Levings, 1990; Harvey, 2004). Use of additional CMS/*Rf* sources would diversify the gene pool of the crop and reduce genetic vulnerability (Leclercq, 1969; Jan and Vick, 2007). The discovery of new CMS sources and corresponding *Rf* genes remains a goal of sunflower breeding.

Downy mildew (DM), caused by *Plasmopara halstedii* (Farlow) Berlese & de Toni, is a major disease affecting sunflower production globally (Gascuel et al., 2015). *P. halstedii* is a biotrophic oomycete pathogen commonly attacks sunflower during seedling stage (Meliala et al., 2000). Host resistance has been considered the best management approach to control the disease. Resistance against *P. halstedii* in sunflower has been described as the typical gene-for-gene interaction (Flor, 1971), where the host resistance *R*-gene(s) recognize and respond to the effector proteins produced by the compatible avirulence (*Avr*) genes of specific pathotypes (Chisholm et al., 2006; Viranyi and Spring, 2011). The DM resistance genes, designated as *Pl*, have long been deployed in elite sunflower lines (Vranceanu and Stoenescu, 1970; Zimmer and Kinman, 1972). However, due to dynamic changes in the pathogen population, new virulent races frequently evolve, which often overcome the effectiveness of the existing *Pl* genes. Fortunately, wild annual sunflower species have proven to be a reliable source of *Pl* genes for DM resistance (Seiler et al., 2017). To date, thirty-four *Pl* genes have been reported in sunflowers, namely *Pl_1_–Pl_33_*, and *Pl_Arg_* (for review see Ma et al., 2017; Dimitrijevic and Horn, 2018; Liu et al., 2018; Pecrix et al., 2018a,b). The ambiguity associated with persistent durability of *R*-gene mediated DM resistance compels the sunflower breeders to discover and deploy new *Pl* genes for sustained sunflower production.

Genetic linkage mapping based on biparental populations has been proven as a robust tool for detecting rarely occurring alleles that have a large effect on the phenotype (Nordborg and Weigel, 2008). Numerous dominant genes in many crop species have been successfully mapped using biparental linkage mapping. In contrast, genome wide association studies (GWAS) are a powerful approach to locate common alleles associated with phenotypes with much higher resolution than linkage mapping because they reflect historical recombination events in broad-based diversity panels (Nordborg and Weigel, 2008). In this study, we report the mapping of the two genes in a sunflower biparental mapping population, a fertility restoration gene, *Rf7*, and a DM resistance gene, *Pl_34_*. We also identified single nucleotide polymorphism (SNP) markers associated with the fertility restoration trait in sunflower lines using a GWAS approach. Finally, the detected significant SNP markers were surveyed in a larger population, which constitutes a global sunflower collection, to identify haplotypes associated with the *Rf1* gene. The findings of this study could be a useful resource for identifying new *Rf* gene(s) and help alleviate the potential genetic vulnerability posed due to the exclusive use of a single CMS/*Rf* system in sunflower hybrid production.

## Materials and Methods

### Plant Materials

#### Bi-Parent Mapping Population

The F_2_ and F_2_-derived F_3_ populations were developed from two F_1_ plants derived from the cross RHA 428/HA 234 to map the *Rf* and *Pl* genes. HA 234 (PI 599778) is an oilseed sunflower maintainer line susceptible to DM, that was released by the USDA-ARS and Texas Agricultural Experiment Station in 1971. RHA 428 (PI 619206) is a male fertility restorer oilseed inbred line resistant to DM, which was selected from the cross RHA 801//RHA 365/PI 413157. PI 413157 is a wild *H. annuus* accession collected in New Mexico in 1974. RHA 428 was released by the USDA-ARS and the North Dakota Agricultural Experiment Station in 2000 as a DM resistant inbred line (Miller et al., 2002). DM tests indicated that RHA 428 is resistant to *P. halstedii* races 334, 700, 710, 714, 730, 734, 735, but susceptible to races 737 and 774 (Gulya, personal communication; Gilley et al., 2016). A total of 408 F_2_ plants were grown in the greenhouse in 2011. Because RHA 428 with CMS PET1 was used as a female parent in the initial cross, 126 F_2_ plants were male sterile, while 328 F_2_ plants were fertile and advanced to F_3_ generation for subsequent phenotypic evaluation for DM resistance and male fertility.

#### Genome-Wide Association Study Panel

A GWAS population of 333 sunflower lines comprised of inbred and advanced breeding lines from the USDA-ARS breeding program and Seeds 2000, now Nuseed Americas Inc., was used to map male fertility restoration. This population includes lines from both oil and confection types and from the two major heterotic groups in cultivated sunflower (Table 1). Among the sunflower lines, 226 were USDA-ARS inbred lines released from 1970 to 2011 and the remaining 107 lines were from Nuseed.

**Table 1:** Sources of sunflower genotypes used in genome-wide association study.

Seed source	Market type		Heterotic group		Total
	Oil	Confection	Restorer	Maintainer	
USDA-ARS	194	32	98	128	226
Nuseed	54	53	56	51	107
Total	248	85	154	179	333


#### Sunflower Evaluation Panel

A total of 548 sunflower genotypes were used as an evaluation panel to investigate the distribution of SNP markers associated with the male fertility restoration genes (Supplementary Table S1). These lines include 238 USDA-ARS released inbred lines (126 maintainer lines and 112 male fertility restoration lines), 63 germplasm lines and 247 plant introduction (PI) lines originally collected from 32 countries, which together capture a large portion of the global diversity present in cultivated sunflower. A total of 222 of the USDA-ARS released sunflower lines were common between the GWAS panel and the sunflower evaluation panel.

### Phenotypic Characterization

#### Male Fertility Restoration

Male fertility evaluation was conducted for the bi-parental population. One hundred seventy-two fertile F_2:3_ families were grown in rows of 30 plants in the field at Fargo, ND, in June 2013. Evaluation of male fertility was conducted at the flowering stage. Plants that produced anthers and shed pollen were considered fertile, whereas those without anthers or pollen were considered sterile. Data on fertility restoration of the GWAS lines was obtained from breeder’s records.

#### Downy Mildew Resistance

Evaluation of DM resistance was conducted in the F_2_-derived fertile F_3_ families. A total of 172 F_3_ families along with the parental lines, RHA 428 and HA 234, were evaluated for resistance to DM in the greenhouse under controlled conditions in June 2013. Thirty seedlings of each F_2:3_ family were inoculated with the North American (NA) *P. halstedii* race 734, a virulent race identified in the United States in 2010 (Gulya et al., 2011) following the method described by Gulya et al. (1991). Sunflower seedlings infected by DM display white sporulation on cotyledons and true leaves in greenhouse tests. Resistant plants exhibit no sporulation.

The results of the F_3_ family tests were used to infer the genotypes of F_2_ plants at the DM resistance locus and male fertility restoration locus. A Chi-squared (χ^2^) analysis was performed to verify whether the observed ratios of segregation for the DM resistance and male fertility in the F_3_ population fit expected models.

### DNA Extraction and Genotyping

#### Bi-Parental Mapping Population

Genomic DNA of the parents and 172 F_2_ progenies derived from the cross RHA 428/HA 234 was extracted from lyophilized tissues using the DNeasy 96 Plant Kit (Qiagen, Valencia, CA, United States). DNA concentrations were measured using a NanoDrop 2000 Spectrophotometer (Thermo Fisher Scientific, Wilmington, DE, United States), and were adjusted to 5 ng μl^-1^ for all samples for polymerase chain reaction (PCR) amplification.

A total of 860 simple sequence repeat (SSR) markers were selected to screen polymorphisms between the two parents (Tang et al., 2002; Yu et al., 2003). Bulked segregant analysis was conducted using polymorphic SSR markers (Michelmore et al., 1991). SSR markers associated with male fertility restoration and DM resistance were assessed in the 172 F_2_ individuals for linkage analysis and mapping. PCR of SSR markers was performed according to Qi et al. (2011), and PCR products were detected using an IR2 4300/4200 DNA Analyzer with denaturing polyacrylamide gel electrophoresis (LI-COR, Lincoln, NE, United States).

Additionally, a total of 58 SNP markers previously mapped to the lower end of LG13 were chosen for marker saturation in the region where male fertility restoration and DM resistance genes were mapped. Twenty-two of these SNPs were selected from Talukder’s map (hereafter referred to as NSA SNP) covering a region of 23.79 cM (Supplementary Table S2, Talukder et al., 2014), and 36 SNPs were selected from Bowers’s map (hereafter referred to as SFW SNP) covering a region of 26.62 cM (Supplementary Table S2, Bowers et al., 2012). Genotyping of most of the selected NSA SNP markers were conducted as described below, while genotyping of the SFW SNPs and a few NSA SNPs was performed using a strategy of converting SNPs into length polymorphism markers (Qi et al., 2015; Long et al., 2017). The primer sequences of the 15 polymorphic SFW SNPs and seven NSA SNPs are presented in Supplementary Table S3. The conditions of the SNP PCR reactions were described by Qi et al. (2015), and PCR products were detected using an IR2 4300/4200 DNA Analyzer with denaturing polyacrylamide gel electrophoresis (LI-COR, Lincoln, NE, United States).

#### GWAS and Sunflower Evaluation Panels

Total genomic DNA was extracted from 40 mg lyophilized young leaves of each sunflower line with the DNeasy 96 Plant Kit (Qiagen Inc., Valencia, CA, United States) using a modified protocol (Talukder et al., 2014). DNA was quantified using the PicoGreen kit (Molecular Probes) according to the kit instructions. Genotyping was carried out at BioDiagnostics, Inc., River Falls, WI, United States, with the custom-built Illumina Infinium chip (Illumina Inc., San Diego, CA, United States) containing 8,723 bi-allelic SNP markers developed by the National Sunflower Association (NSA) SNP consortium (Pegadaraju et al., 2013; Talukder et al., 2014). Automated SNP calling was performed using the cluster algorithm implemented in GenomeStudio v1.0 software (Illumina Inc., San Diego, CA, United States). All data was visually inspected and manually rescored if any errors were evident in the genotype calling.

### Linkage Analysis and Mapping of *Rf* and *Pl* Genes

Construction of LG13 genetic map for RHA 428/HA 234 F_2_ population associated with the *Rf* and *Pl* genes were performed using JoinMap 4.1 software with a maximum likelihood mapping algorithm and Kosambi’s mapping function (Van Ooijen, 2006). A minimum likelihood of odds (LOD) ≥ 3.0 and a maximum distance of ≤ 50 centimorgans (cM) were used to test linkage among markers. The graphical representation of the linkage map was drawn using MapChart 2.2 (Voorrips, 2002).

### Genome-Wide Association Study of Male Fertility Restoration

#### Population Structure and Kinship

The population structure of the GWAS panel was estimated using 681 SNP markers randomly selected from all 17 LGs of the sunflower genome that are spaced at least 1 cM apart. Subpopulation membership of each sunflower line was estimated using STRUCTURE v2.3.4 (Pritchard et al., 2000). An ancestry model that allows population admixture with no *a priori* information was used with a burn-in period of 100,000 iterations followed by 200,000 Markov Chain Monte Carlo (MCMC) iterations for subpopulation numbers (K) ranging from 1 to 10. Five runs for each *K* value were performed and the posterior probability [LnP(D)] was determined for each run. The optimum number of subpopulations was determined from ΔK, the rate of change in LnP(D) between successive *K* values, as proposed by Evanno et al. (2005). Kinship relationships among the lines of the GWAS population (*K* matrix) were derived using 4,630 SNP markers with minor allele frequency (MAF) of ≥ 0.05. The SPAGeDi software v1-5a (Hardy and Vekemans, 2002) was used to estimate a mean kinship coefficient (Loiselle et al., 1995) from SNP marker data, where negative kinship values between lines were set to 0 (Yu et al., 2006).

#### Association Mapping Analysis

Out of the 8,723 SNP markers, data from 5,019 markers were selected to run GWAS analyses because of their known map position in the sunflower genome (Talukder et al., 2014). Imputation of missing genotypes was performed using fastPHASE v1.3 software (Scheet and Stephens, 2006), assuming *K* = 38 clusters with the default settings of the EM algorithm. All marker-trait association tests were run using TASSEL v3.0 standalone (Bradbury et al., 2007). The SNP markers with MAF of ≤ 0.05 were removed from the analyses. Four different GWAS models were tested: first, we examined the association between the phenotype and SNP genotypes in a naïve analysis using the general linear model (GLM), y = Xα + e; second, a GLM analysis was performed that accounted for population structure as a cofactor (GLM*Q*), y = Xα + Qβ + e; third, a mixed linear model (MLM) analysis was performed that considered only kinship relatedness in the model, y = Xα + Kμ + e; and finally, an MLM analysis was performed that accounted for both population structure and kinship relatedness in the model (MLM*Q*), y = Xa + Qβ + Kμ + e (Yu et al., 2006). In the equations, *y* is the phenotype, *X* is the SNP genotype matrix, *α* is the vector of marker effects, *Q* is the population membership assignment matrices for subpopulations in the STRUCTURE analysis, *β* is the vector of subpopulation effects, *K* is the relative kinship matrix determined from the marker data, *μ* is the vector of kinship effects and, *e* is the vector of residual effects. *X*α and *Qβ* represent fixed effects, and *K*μ and *e* represent random effects. Quantile-quantile plots of estimated -log10(*P*) were produced for each model using the R statistical package (R Core Team, 2017) by plotting observed *p-*values of marker–trait associations against the expected *p-*values from the assumption that no association exists between markers and trait. The best fitting GWAS model was chosen by assessing the extent to which the analysis produced more significant results than expected by chance. Genome-wide marker–trait association *p*-values was corrected for multiple testing using 5% false discovery rate (FDR) (Benjamini and Hochberg, 1995). Given the distribution of empirical *p-*values of 4,630 markers, the FDR significance level cut-off corresponded to the *p*-value of 1.57^-04^, which was employed as the threshold for significant marker-trait associations in the GWAS analysis.

## Results

### Molecular Mapping of *Rf* and DM Resistance Genes in RHA 428

#### Phenotypic Assessments

A total of 408 F_2_ plants from the cross RHA 428/HA 234 were grown in the greenhouse in 2011. The F_2_ population segregated in 328 male fertile: 126 male sterile, fitting a single gene model of 3 male fertile: 1 homozygous male sterile (χ^2^ = 1.8355, df = 1, *P* = 0.1755). No seed was obtained from the 126 male sterile F_2_ plants. The F_3_ family tests were performed to identify the F_2_ plants homozygous for DM resistance and male fertility. A total of 172 F_3_ families with good seed set were selected and evaluated in the field for male fertility in the summer of 2013. Fifty-six families were all fertile with no segregation, while 116 families showed segregation for fertility restoration. The segregation ratio fit an expected ratio of 1 homozygous male fertile: 2 heterozygous male fertile (χ^2^ = 0.046512, df = 1, *P* = 0.8292). These data indicated that the male fertility restoration in RHA 428 is controlled by a single dominant gene, designated as *Rf*7.

DM tests of 172 F_3_ families with *P. halstedii* race 734 indicated co-segregation with male fertility restoration. Out of the 56 F_3_ families homozygous for male fertility restoration, 52 were homozygous for DM resistance and four F_3_ families were homozygous for susceptibility. Among the 116 F_3_ families segregating for male fertility, 114 were segregating for DM resistance, while only two were homozygous for DM resistance. The results suggested that the DM resistant gene is linked to the male fertility restoration gene. Since RHA 428 shows a differential specificity against *P. helianthi* races other than the known DM *R* genes (Gilley et al., 2016), this gene in RHA 428 was named as *Pl_34_*.

#### Linkage Map Construction of the *Rf* and *Pl* Genes

**FIGURE 1:**
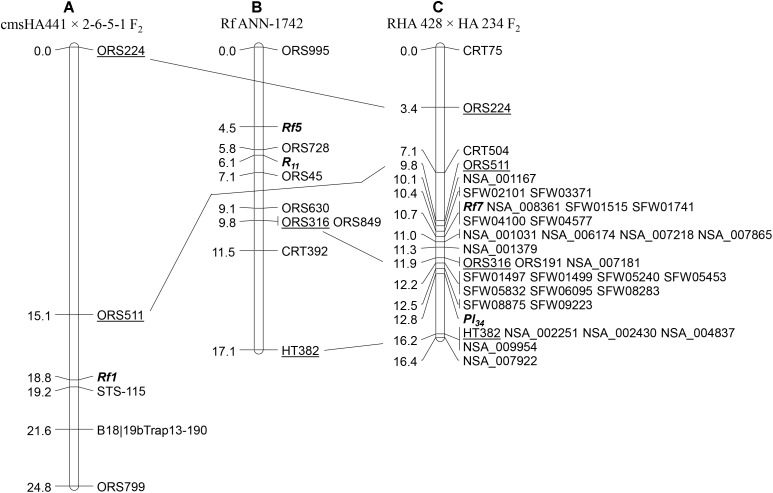
Comparison of the male fertility restoration genes mapped on linkage group 13 in different studies: **(A)** maps showing the location of *Rf1* gene (Yue et al., 2010); **(B)** the location of *Rf5* gene (Qi et al., 2012); and **(C)** the position of *Rf7* gene mapped in the current study. The common markers across different maps are underlined.

Simple sequence repeat markers were used for initial linkage map construction and to study marker-trait association. Out of 860 SSR markers screened for polymorphism between the parents RHA 428 and HA 234, a total of 293 SSR markers (34%) showed polymorphism between two parents. Bulked segregant analysis with polymorphic SSR markers revealed that the male fertility restoration trait was associated with markers on LG13. Seven LG13 specific polymorphic SSR markers were used to screen the F_2_ population, and linkage analysis mapped the *Rf7* gene on LG13 (Figure 1C). The SSR marker, ORS511 mapped at 0.9 cM distal and two co-segregating SSR markers, ORS191 and ORS316 mapped at 1.2 cM proximal to the *Rf7* gene on LG13. The *Pl_34_* DM resistance gene was mapped only 2.1 cM downstream of the *Rf* gene. Two co-segregating SSR markers, ORS191 and ORS316 were 0.9 cM distal to *Pl_34,_* while HT382 was 3.4 cM proximal to *Pl_34_* (Figure 1C).

#### SNP Marker Saturation of the Gene Region

Of the 58 SNP markers tested, 28 showed polymorphism between the two parents, and were assigned to the LG13 map (Figure 1C). Thirteen SNPs were positioned to the *Rf7* gene interval between ORS511 and ORS316, while 14 SNPs were mapped to the *Pl_34_* gene interval between ORS316 and HT382. One was mapped at the end of LG13 (Figure 1C). Out of 13 SNP markers mapped in the *Rf*7 gene interval, five of them were co-segregating with the gene. These five markers spanned 6.9 and 8.9 Mb on chromosome 13 of the XRQ and HA412-HO sunflower genome assemblies, respectively (Table 2).

**Table 2:** Genetic and physical position of *Rf7* and SNP markers linked to *Rf7* in linkage group 13.

NSA map^a^	SFW map^b^	RHA 428 map		Marker/gene	XRQ assembly		HA 412-HO assembly	
cM	cM	cM	No. of recombination		Start (bp)	End (bp)	Start (bp)	End (bp)
	45.1	10.4		SFW03371	169537912	169538031	220170730	220170837
	45.1	10.4	0	SFW02101	169600244	169600352	220283882	220283991
47.8		10.4	0	NSA_001167	170812277	170812673	184040048	184040444
	45.5	10.7	1	SFW01515	170762684	170762803	214069367	214069487
	45.5	10.7	0	SFW04577	170914547	170914666	216832437	216832557
		10.7	0	*Rf7*				
	45.5	10.7	0	SFW04100	172543880	172543768	218716291	218716404
47.6		10.7	0	NSA_008361	175302259	175302630	216316115	216315744
	45.7	10.7	0	SFW01741	177671481	177671385	222970333	222970429
47.7		11.0	1	NSA_006174	178009002	178008648	222929252	222929605
47.7		11.0	0	NSA_007218	178055093	178055296	222887679	222887476
47.7		11.0	0	NSA_007865	178132246	178132059	223268017	223267916
47.9		11.0	0	NSA_001031	178256069	178256386	224145171	224144863
48.8		11.3	1	NSA_001379	181040776	181040397	223557257	223556878
51.5		11.9	2	NSA_007181	184653051	184653455	230738849	230739253


*^a^Marker taken from Talukder et al. (2014), ^*b*^marker taken from Bowers et al. (2012).*

#### Comparison of Other *Rf* Genes Mapped on LG13

Two other male fertility restoration genes, *Rf1* and *Rf5*, have been previously mapped at the lower end of LG13 near the genomic region of the *Rf7*gene mapped in this study (Yue et al., 2010; Qi et al., 2012). The common SSR markers, ORS511 and ORS316 in the *Rf7*map, were separately mapped to the *Rf1* and *Rf5* genetic maps. ORS511 is distal to *Rf1* and *Rf7* with 3.7 and 0.9 cM in their maps, while ORS316 is proximal to *Rf5* and *Rf7* with 5.3 and 1.2 cM in their maps, indicating *Rf7* resides in a *Rf* gene cluster region at the lower end of LG13 (Figure 1).

### Genome-Wide Association Study of the *Rf* Gene

#### Association Analysis

**FIGURE 2:**
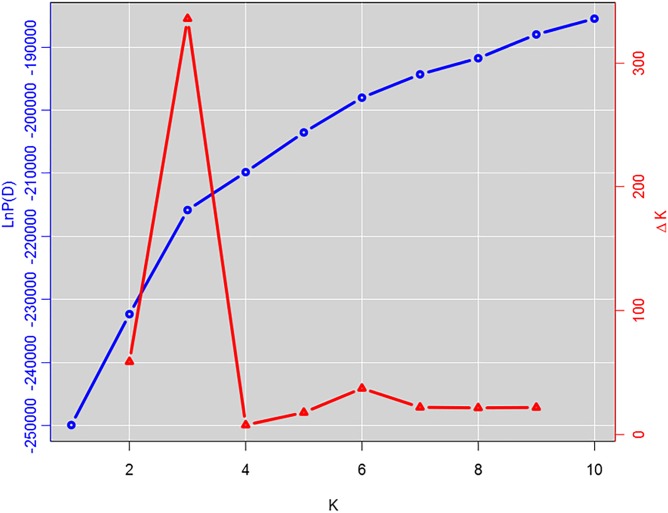
Estimation of number of subpopulations in the genome-wide association study (GWAS) panel. The log probability, LnP(D) for each value of *K* (*K* = 1–10) averaged over 5 runs of STRUCTURE analysis with 100,000 burn-in steps and 200,000 simulation steps is plotted with the Δ*K* values for each of the successive *K* runs.

**FIGURE 3:**
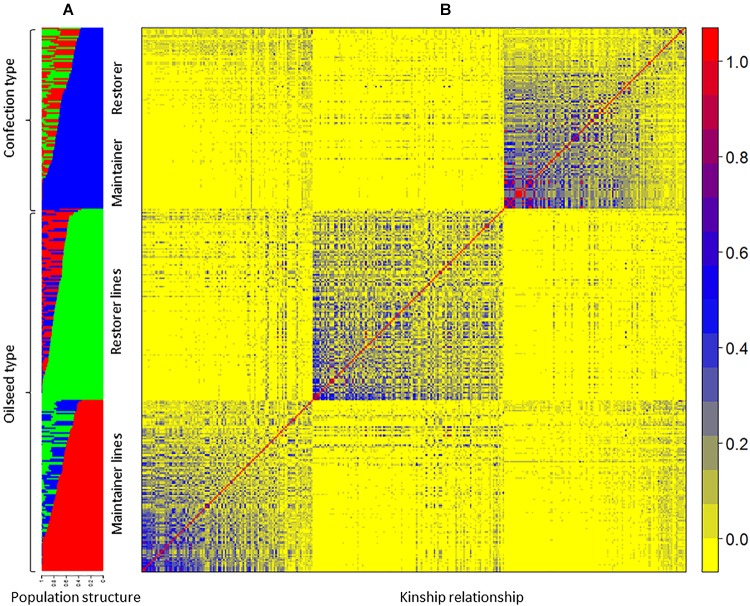
**(A)** Bar plot of the STRUCTURE analysis. Each of the 333 genotypes is represented by a vertical bar, which is partitioned into *K* colored segments that represent the individual’s estimated membership to the *K* clusters, **(B)** heat map of relative kinship matrix among the sunflower lines used in the genome-wide association study panel.

**FIGURE 4:**
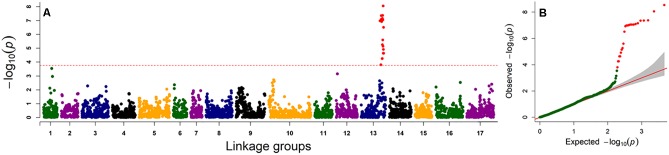
Genome-wide association scan for male fertility restoration trait in sunflower. **(A)** Manhattan plot for male fertility restoration using mixed linear model (MLM*Q*). The dashed horizontal line represents the FDR_0.05_-adjusted significance threshold (*p* < 1.57^-04^), **(B)** Quantile–quantile plots of male fertility restoration trait for MLM*Q* model.

Genome-wide association study analysis was performed using four different models. The best fitting model for this GWAS panel was identified using quantile-quantile plots constructed from the observed vs expected -log_10_(*p*) values of each models (Supplementary Figure S2). As expected, the highest number of significant markers (FDR < 0.05) was observed for the GLM model, with many suspected as false positives. The number of significant markers were dramatically reduced with the inclusion of structure (with *K* = 3 subpopulations; Figure 2, 3) and/or kinship covariates in the model. The deviation of observed *p*-values from the expected *p*-values was minimal for the MLM*Q* model, which accounted both a structure variable and kinship as a random factor. We concluded that this was the best fitting model for our GWAS panel. A total of 24 significant SNP markers were associated with the fertility restoration trait with *p* < 1.57^-04^ (Figure 4). All 24 markers were located from 46.40 to 48.87 cM on LG13 of the NSA sunflower map (Table 3). We performed a blastn search using sequences of the 24 significant SNP markers on both XRQ and HA412-HO sunflower genome assemblies to locate the physical position of these markers^1^. Out of 24 significant SNP markers, no sequence homology was found for NSA_000112 and NSA_007865 on LG13 in either of the sunflower genome assemblies. The remaining 22 significant SNP markers were found between 169361232 and 181040776 bp on chromosome 13 of the XRQ genome (Table 3). This corresponds with 215330128 to 224803649 bp on chromosome 13 of the HA412-HO assembly except for NSA_001167, which was found at the 184040048 bp position on chromosome 13 about 31.29 Mb from the rest of the SNP markers (Table 3).

**Table 3:** Linkage and physical positions of the 24 significant SNP markers associated with the *Rf1* gene identified using genome-wide association mixed model analysis in a panel of 333 sunflower lines.

SNP markers	Talukder’s map (cM)	Alleles		MAF^a^	–log*p*^b^	XRQ assembly physical position			HA412-HO assembly physical position		
						
		Major	Minor			Start (bp)	End (bp)	evalue	Start (bp)	End (bp)	evalue
NSA_003887	46.396	C	A	0.40	6.98	174190882	174191230	2.07^–176^	215331661	215332009	3.0^–176^
NSA_006804	46.396	G	A	0.40	6.98	174189349	174189702	7.64^–166^	215330128	215330481	1.0^–165^
NSA_006551	46.705	C	A	0.46	3.80	170327980	170328401	0	217652288	217652709	0
NSA_009869	46.705	A	G	0.41	7.09	175300674	175301128	0	216317246	216317700	0
NSA_003057	46.801	A	C	0.41	7.35	173358147	173358368	2.33^–87^	220717342	220717563	3.0^–87^
NSA_003720	46.801	A	G	0.41	7.35	173357487	173357807	7.52^–126^	220717903	220718223	1.0^–125^
NSA_000112	46.906	C	A	0.40	7.02	No match	–	–	No match	–	–
NSA_002154	47.273	G	A	0.41	6.92	169595851	169596222	4.73^–143^	220279493	220279864	3.0^–141^
NSA_008247	47.551	G	A	0.42	8.54	174841855	174842376	0	221663595	221664116	0
NSA_006174	47.700	D	I	0.42	4.25	178008648	178009002	0	222929252	222929605	5.0^–149^
NSA_006543	47.700	C	A	0.40	7.05	178055479	178055902	0	222835591	222836014	0
NSA_007218	47.700	A	C	0.44	4.26	178055093	178055296	8.41^–96^	222887476	222887679	1.0^–95^
NSA_007865	47.700	C	A	0.42	5.26	No match	–	–	No match	–	–
NSA_001167	47.820	A	C	0.43	7.15	170812277	170812673	0	184040048	184040444	0
NSA_005156	47.820	G	A	0.41	7.37	169361232	169361457	0	219993535	219993760	1.0^–54^
NSA_001031	47.912	G	A	0.41	5.60	178256069	178256386	3.57^–134^	224144863	224145171	5.0^–134^
NSA_000179	48.018	G	A	0.40	7.05	178008041	178008459	0	222929794	222930212	0
NSA_001915	48.018	G	A	0.40	7.05	177661935	177662387	0	223011979	223012431	0
NSA_008018	48.424	C	A	0.39	5.14	180305755	180306063	3.82^–139^	224803280	224803649	1.0^–170^
NSA_004192	48.587	G	A	0.45	8.05	180994649	180994977	9.18^–157^	223493310	223493664	1.0^–156^
NSA_005572	48.587	A	G	0.38	4.63	180996731	180997065	1.19^–168^	223494955	223495289	2.0^–168^
NSA_007131	48.587	A	G	0.38	4.91	180996014	180996388	0	223494240	223494612	0
NSA_001379	48.755	A	G	0.44	6.51	181040397	181040776	0	223556878	223557257	0
NSA_000042	48.866	A	G	0.38	4.63	180996590	180996915	2.5^–165^	223494814	223495139	3.0^–170^


*^a^Minor allele frequency for each associated marker. ^*b*^Negative logarithm of *p*-value of each associated marker.*

#### Survey of Significant SNP Marker Alleles in the Sunflower Evaluation Panel

Twenty-four significant SNP markers identified in the GWAS analysis were surveyed in the evaluation panel comprised of 548 sunflower lines from the fertility restorer and maintainer heterotic groups, and unassigned PIs. A total of 133 sunflower lines shared all 24 significant SNP marker alleles, of which 92 were USDA released restorer inbred lines, 19 were USDA germplasm lines and another 19 were PI accessions (Table 4 and Supplementary Table S4). Surprisingly, three inbred maintainer lines, HA 452, HA 821 (LP-1), and HA 821 (LS-1), which do not restore fertility in sunflower, also shared all 24 significant SNP marker alleles. Seven restorer lines, RHA 266, RHA 271, RHA 273, RHA 274, RHA 296, RHA 325, and RHA 439, known to possess the *Rf1* gene on LG13, belong to this group of 133 sunflower lines, suggesting that these SNPs are associated with the *Rf1*gene (Supplementary Table S4). In the present study, we mapped the *Rf7* gene from the restorer line RHA 428 on LG13, which shared only seven significant SNP marker alleles detected in the GWAS analysis (Supplementary Table S4). The two inbreed lines, RHA 801 and RHA 365, used as parents in the RHA 428 pedigree shared 24 and 17 significant SNP marker alleles, respectively, different from those of RHA 428 (Supplementary Table S4). Also, the restorer line HA-R9 possessing *Rf5* on LG13, shared only five alleles out of 24 significant SNP marker alleles (Supplementary Table S4). Two restorer inbred lines, RHA 280 and RHA 340, known to possess the *Rf3* gene on LG7, shared only 3–4 significant SNP marker alleles (Supplementary Table S4). Eighteen USDA released restorer inbred lines and three germplasm lines known to restore fertility in sunflower with unknown *Rf* genes shared 2–18 of the significant SNP markers (Table 4 and Supplementary Table S4).

**Table 4:** Distribution of the fertility restoration *(Rf*) gene in the world sunflower collection panel of 548 lines.

No. of sunflower line	*Rf* gene	*Rf* gene LG	No. of shared *Rf1* SNP marker alleles
92 USDA restorer inbred lines	*Rf1*	13	24
19 USDA germplasm lines	*Rf1*	13	24
19 Plant Introduction (PI) lines	*Rf1*	13	24
3 USDA maintainer inbred lines	Unknown	Unknown	24
2 USDA restorer inbred lines	*Rf3*	7	3–4
1 USDA restorer inbred line	*Rf5*	13	5
1 USDA restorer inbred line†	*Rf7*	13	7
21 USDA restorer lines	Unknown	Unknown	2–18
1 line of Rf ARG-420	Unknown	Unknown	0


*†Mapped in RHA 428 in the current study.*

## Discussion

Crop wild relatives (CWR) of sunflower have revolutionized sunflower by providing many genes of utmost economic value, for example, CMS/*Rf* genes for commercial hybrid production, disease resistance genes for rust, DM, Sclerotinia wilt and rot, Phomopsis stem canker, Verticillium wilt, Alternaria leaf spot, and herbicide resistance genes (for review see Seiler et al., 2017). In the present study, we utilized a biparental linkage mapping approach to map the *Rf*7 gene in RHA 428, and an association mapping approach to discover the *Rf1* haplotype and compare it to this and other *Rf* loci. RHA 428 is a progeny derived from the cross involving a wild *H. annuus* accession (PI 413157). Both the male fertility restoration and the DM resistance genes in RHA 428 are derived from PI 413157. Linkage analysis using SSR markers mapped the *Rf7* gene to the lower end of LG13, only 2.1 cM from the *Pl_34_* DM resistance gene (Figure 1C). A pair of co-segregating SSR markers, ORS316 and ORS191, were mapped in between the two genes at 1.2 cM proximal to *Rf7* and 0.9 cM distal to *Pl_34_* (*Rf7*/ORS316 and ORS191/*Pl_34_*). Additional SNP markers selected from two high density SNP maps (Bowers et al., 2012; Talukder et al., 2014) saturated the genomic region around both genes and delimit the genes within even narrower intervals. Five of these SNP markers were co-segregating with the new *Rf7* gene, a useful resource for MAS breeding of fertility restoration (Figure 1C).

### *Rf* Gene Cluster in Sunflower and Other Crops

The lower end of LG13 is very significant for sunflower breeders as many genes of economic importance have been reported to cluster at this genomic region. Qi et al. (2012) mapped a fertility restoration gene, *Rf5* in the wild *H. annuus*-derived Rf ANN-1742 sunflower line, which is tightly linked to the sunflower rust resistance gene *R_11_* (Figure 1B). In the current study, the SSR marker ORS316 mapped at 1.2 cM proximal to *Rf7* also mapped at 5.3 cM proximal to the *Rf5* gene in the Qi et al. (2012) map. The most used male fertility restorer gene, *Rf1*, was also mapped to the lower end of LG13 (Gentzbittel et al., 1995, 1999; Berry et al., 1997; Horn et al., 2003; Yu et al., 2003; Kusterer et al., 2005; Yue et al., 2010). Earlier authors used other DNA markers, including RFLP, amplified fragment length polymorphism (AFLP), random-amplified polymorphism DNA (RAPD) or target region amplification polymorphism (TRAP) markers, to map the *Rf1* gene. Because these markers are not routinely used and can have unclear results, they could not be used for comparative mapping. However, the latest effort of *Rf1* gene mapping (Yue et al., 2010) revealed that an SSR marker, ORS511, mapped at 3.7 cM distal to *Rf1* gene (Figure 1A). The same marker in the current study maps 0.9 cM distal to *Rf7* gene (Figure 1C). Coincident genomic locations of *Rf1*, *Rf5*, and *Rf7* are a strong indication that these genes are clustered on the lower end of LG13. Clustering of *Rf* genes has been observed in other species, for example, four fertility restorers, *Fr*, *Fr*2, *Fr_PI207228_*, and *Fr_XR235_* were mapped to the same linkage group in common bean (Jia et al., 1997), in rice *Rf1a*, *Rf1b*, *Rf4* and *Rf5*, which encode a PPR protein, are clustered on chromosome 10 near the *Rf1* locus (Zhang et al., 2002; Akagi et al., 2004; Komori et al., 2004; Wang et al., 2006; Fujii et al., 2008; Hu et al., 2012; Kazama and Toriyama, 2014; Melonek et al., 2016). PPR-gene clusters have also been reported at petunia *Rf* (Bentolila et al., 2002), radish *Rf*o/*Rfk1* (Brown et al., 2003; Koizuka et al., 2003) and, *Rf1* and *Rf2* of monkeyflower (*Mimulus guttatus*) on LG7 (Barr and Fishman, 2010).

### Relationship of the *Rf* Genes in the LG13 Cluster

The male fertility restorer gene *Rf1* was discovered in the sunflower line T66006-2-1, derived from a cross involving a wild *H. annuus* accession from Texas (Supplementary Figure S1). The other two *Rf* genes, *Rf5* and *Rf7*, mapped in the same LG13 were derived from sunflower lines developed using two independent wild *H. annuus* accessions. The *H. annuus* accession of the *Rf5* gene (PI 613748) was collected from Oklahoma, while the wild accession of the *Rf7* gene (PI 413157) in our study was a collection from New Mexico, United States. In the present study, 24 significant SNP markers were identified to be associated with the *Rf1* gene. Of the 159 restorer lines in an evaluation panel of 548 sunflower lines, 130 lines retained all 24 SNP alleles. This finding is consistent with previous reports of widespread introduction of the dominant nuclear restorer *Rf1* gene in the sunflower breeding materials (Korell et al., 1992; Serieys, 2005; Jan and Vick, 2007). It seems that 24 significant SNP alleles associated with *Rf1* were transmitted as a haplotype for over five decades (1970–2011) of breeding and the development of 92 restorer lines (Table 4 and Supplementary Table S4). In addition, 19 restorer germplasm lines with restoration from different wild species and 19 PI lines collected from 10 different countries also retained 24 significant SNP alleles. However, in the comparison of 24 SNP marker alleles associated with the *Rf1* gene to HA-R9 (*Rf5*) and RHA 428 (*Rf7*), only five and seven SNPs retained the *Rf1* alleles in HA-R9 and RHA 428, respectively (Supplementary Table S4). In addition, one NSA SNP marker, NSA_008361 co-segregating with *Rf7* identified in the present study was not found to be associated with *Rf1* in GWAS analysis (Table 3). *Rf5* is linked to a rust *R* gene *R_11_*, while *Rf7* is linked to a DM *R* gene *Pl_34_* (Figure 1B,C). Taken together, it indicated that the *Rf*5 and *Rf*7 genes are different in genomic composition than the *Rf1* gene. However, we cannot rule out the possibility that *Rf*7 could potentially be an alternate *Rf1* source to that of the original wild *H annuus* in Texas. Further characterization of these closely linked genes would elucidate the evolutionary relationships among *Rf1*, *Rf5*, and *Rf7* by additional fine mapping combined with a whole genome resequencing approach.

Due to the size and the complexity of the sunflower genome, cloning of sunflower *Rf* genes has not been successful yet. Owens et al. (2018) recently reported a candidate gene, HanXRQChr13g0419821 which encodes an aldehyde dehydrogenase gene for *Rf1*. HanXRQChr13g0419821 is located at the 174,082,899 bp position within the interval of 24 significant SNPs associated with *Rf1* (Table 3). Meanwhile, map and sequence-based analysis of the *Rf5* gene region on HA412-HO and XRQ genome assemblies identified two candidate genes for *Rf5*, which encodes PPR proteins (Qi et al., 2018).

Surprisingly, three USDA-ARS released inbred lines, HA 452, HA 821 (LP-1) and HA 821 (LS-1) also shared all 24 SNP marker alleles associated with *Rf1*. These inbred lines are maintainer lines with no *Rf* allele. HA 452 is an F_6_ derived F_7_ line selected from the cross of two maintainer lines, HA 335/HA 412 (Miller et al., 2006). HA 821 (LP-1) and HA 821 (LS-1) were derived from mutagenesis of a maintainer line, HA 821 (Miller and Vick, 1999). While the pedigree of these sunflower lines clearly suggests that these are maintainer lines, the exact reason for the presence of positive SNP alleles associated with *Rf1* gene is not known.

### A Strategy for Using *Pl_34_* With *Rf7* in Sunflower Breeding

In this study, we also mapped a DM resistance gene, *Pl_34,_* at the lower end of LG13 that is tightly linked to the *Rf7* fertility gene, at a genetic distance of 2.1 cM. Three additional DM resistance genes, *Pl_5_*, *Pl_8_* and *Pl_21_*, have been previously reported to map in this genomic region (Bert et al., 2001; Radwan et al., 2003; Bachlava et al., 2011; Vincourt et al., 2012; Qi et al., 2017). Comparative analysis of map location implies that *Pl_34_*, *Pl_8_*, *Pl_5_*, and *Pl_21_* are positioned at 0.9, 1.2, 5.1, and 13.2 cM proximal to the common SSR marker, ORS316 (Vincourt et al., 2012; Qi et al., 2017), suggesting that *Pl_21_* is little farther away from the remaining three-gene in the cluster. The *Pl*_5_ and *Pl*_8_ genes originated from wild *H. tuberosus* and *H. argophyllus*, respectively, while the *Pl_34_* was derived from wild *H. annuus*. In addition to their diverse origin, *Pl_5_*_,_ *Pl_8_*_,_ and *Pl_34_* also showed differential response against a recent collection of 185 *P. halstedii* isolates in the United States (Gilley et al., 2016). Two diagnostic SNP markers, NSA_000423 and NSA_002220, for *Pl_8_* were not mapped in the *Pl_34_* map (Qi et al., 2017). Taken together, this indicates that *Pl_34_* is a different gene from *Pl*_5_ and *Pl*_8_. It appears that *Pl_34_* might not be a good choice as a sole defense against the damage caused by the DM but could be a good candidate for pyramiding *Pl* resistance in sunflower lines that could preferentially be transferred along with the tightly linked *Rf7* fertility restoration gene identified in this study. The *Rf1* gene has been extensively used in sunflower breeding over four decades for hybrid seed production. The use of the new *Rf7* gene would potentially diversify the genetic makeup of the hybrids. The high-throughput amenable SNP markers co-segregating with the *Rf7* gene will expedite MAS and transfer of the *Rf* allele into elite breeding lines through conventional breeding.

## Data Availability

The datasets generated for this study can be found in the publicly accessible repository: figshare (https://doi.org/10.6084/m9.figshare.7754378.v1 and https://doi.org/10.6084/m9.figshare.7754420.v1).

## Ethics Statement

The experiments were performed in compliance with current laws of the United States.

## Author Contributions

LQ, BH, and C-CJ conceived and designed the experiments. All authors performed the experiments. ZT and LQ analyzed the data and wrote the paper. BH, GM, and C-CJ commented on the manuscript before submission.

## Conflict of Interest Statement

The authors declare that the research was conducted in the absence of any commercial or financial relationships that could be construed as a potential conflict of interest.

## Footnotes

1 https://www.heliagene.org

## Supplementary Material

Figure S1 Pedigree of the *Rf1* gene origin (taken from Korell et al., 1992).

Figure S2 Quantile–quantile plots of observed vs. expected –log_10_ (*p*) values from four different genome-wide association models: **(i)** general linear model (GLM), **(ii)** general linear model with population structure used as a cofactor (GLM*Q*), **(iii**) mixed linear model that accounted only kinship relatedness (MLM), and **(iv)** a mixed linear model that accounted for both population structure and kinship relatedness in the analysis (MLM*Q*). The red lines represent the expected values under the null distribution.

Table S1 List of sunflower inbred/germplasm lines used for single nucleotide polymorphism marker validation associated with male fertility restoration Rf1 gene. The detailed information of each line can be found in the USDA National Plant Germplasm Systerm: https://www.ars-grin.gov/npgs/.

Table S2 Map position and sequences of SNP markers on LG13 selected from published sunflower linkage maps used for map saturation in the present study.

Table S3 Allele-specific polymerase chain reaction (PCR) primer sequences of polymorphic SNP markers mapped in this study.

Table S4 Distribution of 24 significant SNP markers in the sunflower evaluation panel identified using GWAS analysis of fertility restoration trait. The black font color indicates SNP allele associated with fertility restoration, while the red font color indicates the alternate SNP allele.
