# Supplementary figures and images for: Linkage Mapping and Genome-Wide Association Studies of the Rf Gene Cluster in Sunflower (Helianthus annuus L.) and Their Distribution in World Sunflower Collections
Source: Front Genet. 2019 Mar 14;10:216. doi: 10.3389/fgene.2019.00216 (PMC6426773; doi:10.3389/fgene.2019.00216)

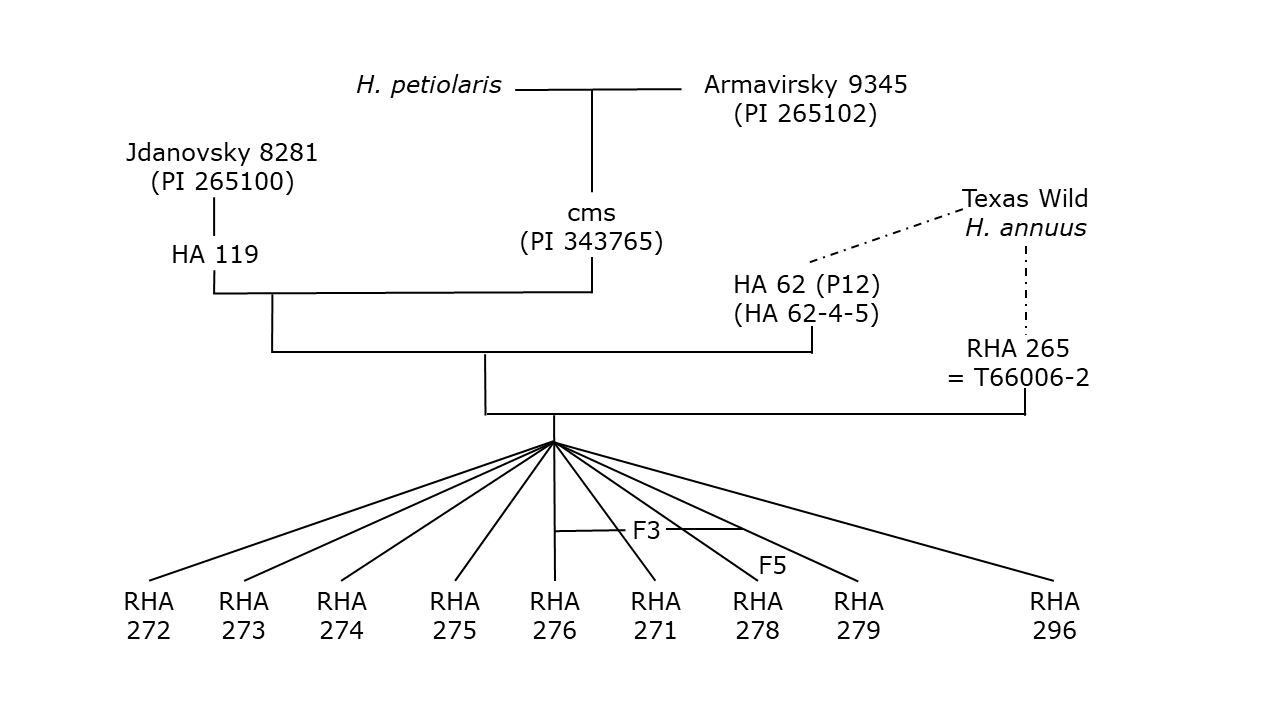

Supplement: Figure S1 — Pedigree of the Rf1 gene origin (taken from Korell et al., 1992). [file Image_1.TIF]

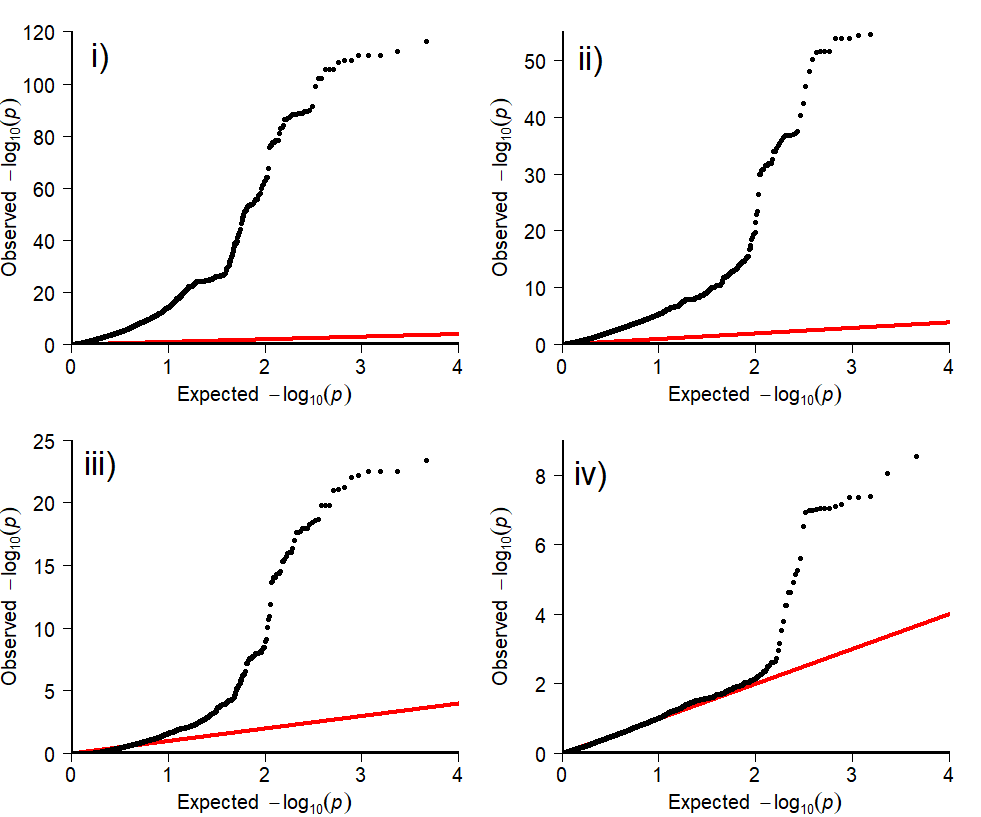

Supplement: Figure S2 — Quantile–quantile plots of observed vs. expected –log10 (p) values from four different genome-wide association models: (i) general linear model (GLM), (ii) general linear model with population structure used as a cofactor (GLMQ), (iii) mixed linear model that accounted only kinship relatedness (MLM), and (iv) a mixed linear model that accounted for both population structure and kinship relatedness in the analysis (MLMQ). The red lines represent the expected values under the null distribution. [file Image_2.TIF]
